# Prevalence of *Naegleria fowleri* in Environmental Samples from Northern Part of India

**DOI:** 10.1371/journal.pone.0137736

**Published:** 2015-10-20

**Authors:** Ashutosh Panda, Shehla Khalil, Bijay Ranjan Mirdha, Yogita Singh, Samander Kaushik

**Affiliations:** 1 Department of Microbiology, All India Institute of Medical Sciences, New Delhi, India; 2 Centre for Biotechnology, Maharishi Dayanand University, Rohtak, Harayana, India; NERC Centre for Ecology & Hydrology, UNITED KINGDOM

## Abstract

*Naegleria fowleri* the causative agent of Primary Amoebic Meningoencephalitis, is ubiquitously distributed worldwide in various warm aquatic environments and soil habitats. The present study reports on the presence of *Naegleria* spp. in various water bodies present in Rohtak and Jhajjar district, of state Haryana, India. A total of 107 water reservoirs were screened from summer till autumn (2012 and 2013). In order to isolate *Naegleria* spp. from the collected water samples, the water samples were filtered and the trapped debris after processing were transferred to non-nutrient agar plates already seeded with lawn culture of *Escherichia coli*. Out of total 107 water samples, 43 (40%) samples were positive by culture for free living amoeba after incubation for 14 days at 37°C. To identify the isolates, the ITS1, 5.8SrDNA and ITS2 regions were targeted for PCR assay. Out of total 43 positive samples, 37 isolates were positive for *Naegleria* spp. using genus specific primers and the most frequently isolated species was *Naegleria australiensis*. Out of 37 *Naegleria* spp. positive isolates, 1 isolate was positive for *Naegleria fowleri*. The sequence analysis revealed that the *Naegleria fowleri* strain belonged to Type 2.

## Introduction

Free-living amoebae (FLA) such as *Naegleria*, *Acanthamoeba*, *Vahlkampfia* and *Hartmannella* are ubiquitously distributed worldwide in various aquatic and soil habitats. Many species of the genera *Naegleria* are known based on the analysis of their small subunit ribosomal deoxyribonucleic acid (SSU rDNA), large subunit ribosomal deoxyribonucleic acid (LSU rDNA) and the internal transcribed spacer (ITS) regions, including 5.8S rDNA [1]. *Naegleria fowleri* (*N*. *fowleri*) is the only species pathogenic to humans; it causes primary amoebic meningoencephalitis (PAM) [2–4]. PAM is rare but almost always fatal disease of the central nervous system (CNS) [4–7] often reported in healthy children and young adults after exposure to contaminated recreational, domestic or environmental water sources [4–6]. *N*. *fowleri* is thermophilic organism and can tolerate temperatures up to 45°C. Hence, these amoebae proliferate during warmer months of the year when the ambient temperature is likely to be high. *N*. *fowleri* has been isolated from various aquatic habitats such as swimming pools, lakes, rivers, hot springs and tap water. Distribution of FLA including *N*. *fowleri* has been reported to range between 23% and 89% in some geographical locations [8–18]. Accidental exposure and infections occur primarily in children and young adults–as they are more active in aquatic activities (diving, jumping into water and underwater swimming) and are likely to come into direct contact with free living amoebae in contaminated water. The organism enters the nasal cavity and migrates through the olfactory neuro-epithelium to the central nervous system and causes a fatal infection that exhibits symptoms similar to acute bacterial meningitis.

There are at least 235 reported PAM cases worldwide [19], so the disease can be viewed extremely rare. But as it is almost always fatal, only about 5% of patients survive. Nearly two third cases of PAM have been reported from the United States of America (USA) alone and rest from the other parts of the world. So far, there are only 16 reported cases of PAM in India [20–35]. Out of 16 cases only 4 patients survived, of which 3 had no history of exposure to recreational water bodies, which makes it again suspicious whether these cases truly were *N*. *fowleri* infections. Of remaining 12 patients, 11 patients expired due to the disease, of which 4 were infants below 6 months of age. No conclusive information could be established for one patient as it was lost to follow up [31]. The presence of *N*. *fowleri* in swimming pools [36], pond water [37] and sewage canals [38] in India has been confirmed. It is believed that neurological infections by these free-living amoebas are misdiagnosed as well as under reported in India, probably due to inadequate information regarding their pathology or due to a very low autopsy rate.

It is also intriguing to note that above mentioned Indian reports are primarily case reports based on direct demonstration of *N*. *fowleri* in the CSF of the infected individual. However, no information was available on the source of infection as well as molecular detection of *Naegleria* species in environmental samples. In view of this the aim of the present study was to investigate the distribution of FLA with special reference to *N*. *fowleri* in selected water bodies in Rohtak and Jhajjar, of state Haryana, India. These two sites of the state were judiciously selected because of the fact that most of the clinical cases in North India were reported from these parts of the state.

## Material and Methods

No ethical permission was required to carry out the study. The samples which were collected from the various water bodies didn't require any prior permission from civic agencies of the state. One of the co-author, Dr. Samander Kasuhik of Maharishi Dayanand University, Rohtak, Haryana had taken prior permission from the local leaders of the various villages for this study.

### Sampling sites and sample collection

Collection of samples from selected water bodies in Rohtak and Jhajjar of state Harayana were done every 14 days from summer till autumn during the year 2012 and 2013. A total of 107 water reservoirs including ponds and lakes were screened. 5 samples from each of the 107 sites were collected by immersion of a 100 ml Schott Duran bottle into the upper 2 cm of the respective water body. Prior to sample collection, temperature and pH of the sampling sites were measured for each sample taken. The water samples were transported to the laboratory at ambient temperature to maintain the thermophilic amoeba in natural conditions and were processed within 2–4 hrs after sampling.

### Isolation and culturing of trophozoites

In order to isolate free living amoeba *Naegleria* spp. from the collected water samples, further processing of the samples were done. The five, 100 ml volumes that were collected from different parts of a single source was composited to make a 500 ml volume for the analysis. The samples were further filtered through a nitrocellulose membrane (pore size, 0.22 μm, Millipore, Fisher Scientific) [39]. Two to Eight nitrocellulose membranes were used to filter 500ml of water. The number of nitrocellulose membrane used for each sample varied on the basis of turbidity of the water sample. After filtration, the nitrocellulose membranes containing the trapped debris were flushed carefully *in situ* with 10 ml of sterile distilled water [40]. The water containing the debris after flushing was not possible to examine microscopically due to heavy content of the trapped soil and other impurities. However, it was further processed by centrifugation for 15mins at 1200 rpm. The pellet obtained was further re-suspended in 100 μl of supernatant.

Re-suspended pellets were gently pipetted onto a non-nutrient agar (NNA) plates prepared from PAGE amoeba saline overlaid with a lawn culture of *Escherichia coli*. The culture plates were sealed with parafilm and incubated at 37°C for upto 14 days [40, 41]. A temperature of 42–45°C is preferably used for the isolation of *Naegleria* species. However, to avoid the possible prohibitive effect of higher temperatures on the growth of certain non thermotolerant free-living amoebae a temperature of 37°C was selected in the present study [42].

### Detection of *Naegleria* species

Daily examinations of all the culture plates were done up to 14 days using a light microscope (Olympus CX31). Cultures lacking morphological features of amoeba within 14 days were deemed negative and discarded. On initial observation it was found that the culture plates were colonized with mixed organisms and FLA like organisms. The culture plates which were suspected to be positive based on morphological characters were further subjected to clone establishment of the trophozoites by means of migration method [43]. Briefly, a block of agar containing small numbers of amoeba was transferred to a fresh NNA plate prepared from PAGE amoeba saline overlaid with a lawn culture of *Escherichia coli*. For documentation morphological characteristics were photographed.

### PCR assays and DNA sequencing

The trophozoites were gently scraped from agar plates, re-suspended in PBS and subsequently pelleted by sedimentation at 1000g for 10 min. Total genomic DNA was extracted from amoeba trophozoites using the QIAamp DNA mini kit (Qiagen, Hilden, Germany). The positive control for the standardization of the PCR assay was isolated from *N*. *fowleri* culture obtained from American Type Culture Collection (ATCC no. 30894).

Fwl, (5'-GTGAAAACCTTTTTTCCATTTACA-3') and RV1, (5'-AAATAAAAGATTGACCATTTGAAA-3') were used as forward and reverse primers respectively for *N*. *fowleri*. Fw2, (5’- GAACCTGCGTAGGGATCATTT -3’) and RV2, (5’- TTTCTTTTCCTCCCCTTATTA -3’) were used as forward and reverse primers respectively for genus *Naegleria*. These primers are located respectively in the ITS1 and ITS2 regions [44]. Amplification was carried out with a PCR buffer containing 10 mMTrisHCl pH 9, 50 mM KCI, 1.5 mMMgCl_2_, 0.1% Triton X 100 and 0.24 mM of each dNTPs, 0.4 μM of each primer, 1 unit of Taq DNA polymerase and 50–100 ng of genomic DNA in a final volume of 25μl. The PCR conditions comprised of initial denaturation for 3 min at 94°C, followed by 30 cycles at 94°C for 30 s, 55°C for 30 s and 72°C for 30 s with a final elongation step at 72°C for 5 min. The expected PCR amplified product for *Naegleria* genus was 408–457 bp and for *N*. *fowleri* it was 310bp. The product was analysed by electrophoresis on 1.5% agarose stained with ethidium bromide and was visualized on a UV transilluminator. For the PCR assay described in this study a positive reaction was obtained with 9pg of purified DNA but not with the subsequent dilution tested of 0.9pg. The specificity of the primers was checked with BLAST for other meningitis causing organisms like *Cryptococcus neoformans*, *Haemophilus influenzae*, *Neisseria meningitidis*, *Staphylococcus aureus*, *Escherichia coli*, *Mycobacterium tuberculosis*, *Candida albicans* and *Acanthamoeba castellanii* **.** No cross reactivity was observed with any of the above mentioned organisms.

### Nucleotide Sequencing

The amplicons from 10 randomly selected isolates were gel purified using the QIAquick gel extraction kit (Qiagen, Hilden, Germany). These were directly sequenced in both directions using respective forward and reverse primers using the BigDye Terminator sequencing v3.1 kit and an ABI 3130 XL genetic analyzer (Applied Biosystems, Foster City, CA, USA).

DNA chromatograms were examined using the BioEdit software version 7.1.3. The forward and reverse sequences were pair-wise aligned using ClustalW software and were manually refined to obtain a better consensus sequence. For nucleotide similarities a homology search was performed with BLAST against all eukaryotic nucleotide sequences archived in the GenBank database. Nucleotide residues corresponding to primer regions were excluded from the similarities search analyses.

### Nucleotide Sequence Accession Number

The nucleotide sequences determined from this study were submitted to the Genbank and were assigned accession numbers KF700040, KF709528, KF709529, KF709530, KF709531, KF709532, KF709533, KF709534, KF709535 and KF709536.

### Phylogenetic analysis

Phylogenetic analysis was carried out based on the internal transcribed spacers (ITS) and 5.8S sequences using the Molecular Evolutionary Genetics Analysis (MEGA) software. Phylogenetic trees were constructed using the neighbour joining with molecular distances estimated by the Kimura two-parameter model. The bootstrap procedure was then used to evaluate the robustness of each node.

## Results

### Microscopy and PCR assay result

**Fig 1:**
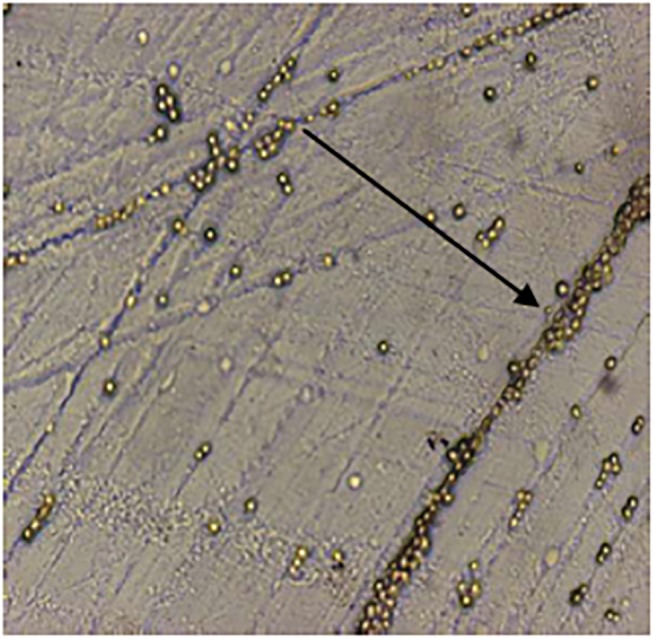
Morphological observation of *Naegleria* -like cyst at 100X magnification. Zone of *Escherichia coli* clearing progressed and the number of cysts increased markedly.

**Fig 2:**
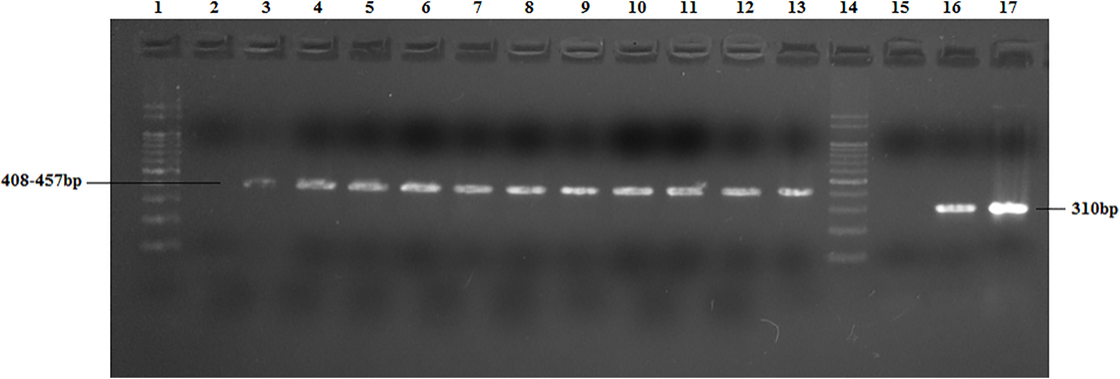
Amplicon bands revealed by genus and species specific primers against *Naegleria* isolates for representative samples. Genus *Naegleria* specific amplification (Lane 1–13), Lane 1 Ladder 100bp, Lane 2 Negative control, Lane 3 Positive control, Lane 4–13 Positive samples. *N*. *fowleri* specific amplification (14–17), Lane 14. Ladder 100bp, Lane 15 Negative control, Lane 16 Positive control, Lane 17 Positive sample.

A total of 107 water reservoirs including ponds and lakes were screened. The temperature, pH of these water reservoirs ranged between 24–31°C and 5.8–7.2 pH respectively (Table 1). Out of total 107 water samples tested, 43 (40%) samples were positive for free living amoeba by culture. Cultures were examined microscopically for the presence of morphological forms in all 43 isolates (Fig 1). These positive isolates were further subjected to PCR assay for detection of genus (*Naegleria*) as well as species (*N*. *fowleri*). Out of total 43 microscopically suspected positive samples, 37 (86%) isolates showed positive amplicons for genus *Naegleria*; where as 1 out of 37 (3%) amplicons was positive for *N*. *fowleri* (Fig 2).

**Table 1:** Overview of geographic sites sampled for *Naegleria* spp.

Month	Sample ID	Geographical Coordinates of Sampling Sites	Temperature	pH	PCR for *Naegleria*
March	R1CV	29^0^03’27”N 76^0^32’18”E	29°C	7.6	-
	R2CV	29^0^03’43”N 76^0^31’46”E	30°C	7.1	+
	R3LKM	29^0^02’19”N 76^0^28’34”E	28°C	7.5	-
	R4LKM	29^0^02’38”N 76^0^28’26”E	29°C	7.3	-
	R5LKM	29^0^02’43”N 76^0^28’40”E	29°C	7.1	-
	R6CHV	29^0^01’37”N 76^0^30’01”E	27°C	7.4	-
	R7CHV	29^0^01’53”N 76^0^30’17”E	26°C	7.6	-
	R8CHV	29^0^01’59”N 76^0^30’09”E	27°C	7.1	-
	R9BHW	28^0^59’13”N 76^0^30’49”E	29°C	5.8	+
	R10BHW	28^0^59’10”N 76^0^31’05”E	30°C	5.9	+
	R11BHW	28^0^59’06”N 76^0^31’10”E	29°C	5.8	-
	R12KIL	28^0^56’54”N 76^0^42’44”E	30°C	6.2	-
	R13KIL	28^0^56’44”N 76^0^42’38”E	29°C	6.5	-
	R14KIL	28^0^56’54”N 76^0^43’14”E	29°C	6.3	-
	R15KIL	28^0^56’30”N 76^0^42’57”E	29°C	6.7	-
April	R16BHT	28^0^54’25”N 76^0^42’19”E	30°C	5.9	+
	R17BHT	28^0^54’24”N 76^0^41’57”E	29°C	6.0	+
	R18BHT	28^0^54’11”N 76^0^41’56”E	29°C	5.8	-
	R19RHC	28^0^54’14”N 76^0^34’25”E	28°C	7.1	-
	R20RHC	28^0^54’37”N 76^0^34’11”E	29°C	7.2	-
	R21RHC	28^0^54’01”N 76^0^34’01”E	28°C	6.8	-
	R22MOK	28^0^53’09”N 76^0^25’50”E	28°C	6.4	+
	R23MOK	28^0^53’22”N 76^0^25’45”E	29°C	6.7	+
	R24MOK	28^0^53’29”N 76^0^25’45”E	29°C	5.9	+
	R25BOH	28^0^53’38”N 76^0^38’42”E	28°C	6.2	-
	R26BOH	28^0^53’50”N 76^0^39’37”E	29°C	6.4	-
	R27BAL	28^0^52’48”N 76^0^41’09”E	29°C	6.7	+
	R28BAL	28^0^52’02”N 76^0^41’28”E	30°C	6.9	-
	R29BAL	28^0^52’35”N 76^0^41’33”E	29°C	6.7	-
	R30BAL	28^0^52’32”N 76^0^41’25”E	29°C	6.3	-
	R31SKK	28^0^51’43”N 76^0^34’10”E	28°C	6.6	-
	R32SKK	28^0^51’42”N 76^0^33’54”E	30°C	6.8	-
	R33SKK	28^0^51’30”N 76^0^34’22”E	29°C	5.9	-
May	R34KSV	28^0^51’30”N 76^0^40’43”E	29°C	6.8	+
	R35KSV	28^0^51’42”N 76^0^40’16”E	30°C	6.9	+
	R36KSV	28^0^51’18”N 76^0^39’58”E	30°C	6.1	+
	R37MAV	28^0^51’01”N 76^0^36’17”E	29°C	6.4	-
	R38MAV	28^0^50’38”N 76^0^36’13”E	29°C	6.3	-
	R39GRW	28^0^49’12”N 76^0^33’46”E	28°C	6.1	+
	R40KAL	28^0^49’40”N 76^0^23’22”E	29°C	5.9	-
	R41KRT	28^0^48’11”N 76^0^37’23”E	30°C	6.9	+
	R42KRT	28^0^48’11”N 76^0^36’53”E	30°C	7.1	+
	R43KRT	28^0^47’48”N 76^0^37’01”E	29°C	7.0	+
	R44KRT	28^0^47’50”N 76^0^37’25”E	30°C	6.8	-
	R45SAP	28^0^46’28”N 76^0^46’31”E	30°C	6.5	-
	R46SAP	28^0^46’47”N 76^0^46’33”E	30°C	6.7	+
	R47SAP	28^0^46’40”N 76^0^45’41”E	31°C	6.8	-
	R48SAP	28^0^46’06”N 76^0^46’08”E	30°C	6.5	+
	R49SAP	28^0^46’09”N 76^0^46’25”E	31°C	6.8	+
June	R50GRI	28^0^45’44”N 76^0^46’17”E	30°C	6.3	-
	R51GRI	28^0^45’30”N 76^0^46’20”E	30°C	6.3	+
	J52DIG	28^0^46’12”N 76^0^37’43”E	29°C	5.9	-
	J53DIG	28^0^46’17”N 76^0^38’03”E	30°C	5.8	-
	J54DIG	28^0^45’57”N 76^0^37’36”E	29°C	6.0	+
	J55DIG	28^0^45’50”N 76^0^38’10”E	29°C	6.2	-
	J56DIG	28^0^45’43”N 76^0^38’03”E	30°C	5.9	+
	J57DIG	28^0^45’33”N 76^0^37’42”E	30°C	6.7	+
	J58DHA	28^0^45’22”N 76^0^37’12”E	30°C	6.1	-
	J59DHA	28^0^45’14”N 76^0^37’20”E	31°C	6.3	-
	J60DHA	28^0^45’10”N 76^0^37’12”E	30°C	6.6	-
	J61GOC	28^0^44’02”N 76^0^35’41”E	31°C	5.9	+
	J62GOC	28^0^44’05”N 76^0^36’00”E	30°C	5.8	+
	J63SHE	28^0^43’17”N 76^0^36’34”E	31°C	6.5	+
	J64SHE	28^0^43’12”N 76^0^36’34”E	29°C	6.0	-
	J65SHE	28^0^43’05”N 76^0^36’30”E	29°C	6.1	-
	J66SHE	28^0^43’14”N 76^0^37’03”E	30°C	6.3	+
	J67SHE	28^0^42’47”N 76^0^36’37”E	29°C	6.6	+
	J68SHE	28^0^42’59”N 76^0^37’02”E	31°C	6.4	+
July	J69MAN	28^0^42’35”N 76^0^39’02”E	29°C	5.9	-
	J70MAN	28^0^42’46”N 76^0^38’34”E	28°C	5.8	-
	J71MAN	28^0^43’04”N 76^0^38’39”E	29°C	6.0	-
	J72MAN	28^0^43’03”N 76^0^39’06”E	29°C	6.3	-
	J73CHC	28^0^43’38”N 76^0^40’20”E	30°C	6.1	-
	J74CHC	28^0^43’42”N 76^0^40’30”E	31°C	5.9	-
	J75CHC	28^0^43’36”N 76^0^40’40”E	30°C	6.3	-
	J76CHC	28^0^43’34”N 76^0^40’46”E	29°C	6.2	-
	J77CHC	28^0^43’27”N 76^0^40’31”E	30°C	6.7	-
	J78CHC	28^0^41’01”N 76^0^36’56”E	30°C	6.4	-
	J79CHC	28^0^40’40”N 76^0^37’25”E	29°C	5.9	-
	J80BER	28^0^41’56”N 76^0^34’55”E	30°C	6.0	+
	J81BER	28^0^41’41”N 76^0^34’39”E	31°C	5.8	+
	J82BER^#^	28^0^41’55”N 76^0^34’26”E	31°C	6.3	+
	J83BER	28^0^42’16”N 76^0^34’28”E	30°C	6.7	-
	J84BER	28^0^42’25”N 76^0^34’49”E	31°C	6.2	+
	J85BER	28^0^42’34”N 76^0^34’31”E	29°C	6.4	+
	J86BER	28^0^41’42”N 76^0^34’06”E	30°C	6.8	-
	J87BAG	28^0^41’30”N 76^0^31’51”E	27°C	7.1	-
	J88BAG	28^0^41’18”N 76^0^33’42”E	27°C	7.0	-
	J89WAZ	28^0^40’53”N 76^0^34’21”E	28°C	7.2	-
	J90WAZ	28^0^40’51”N 76^0^34’35”E	27°C	6.9	+
	J91WAZ	28^0^40’59”N 76^0^34’33”E	27°C	7.0	+
August	J92MAJ	28^0^40’51”N 76^0^27’06”E	24°C	7.2	-
	J93MAJ	28^0^40’14”N 76^0^27’01”E	25°C	7.0	+
	J94MAJ	28^0^40’13”N 76^0^27’08”E	24°C	7.1	-
	J95MAJ	28^0^40’18”N 76^0^27’08”E	25°C	7.1	-
	J96MAJ	28^0^40’21”N 76^0^27’16”E	24°C	6.9	-
	J97MAJ	28^0^40’15”N 76^0^27’32”E	24°C	6.9	-
	J98MAJ	28^0^40’25”N 76^0^27’12”E	25°C	7.2	-
	J99MAJ	28^0^40’28”N 76^0^27’59”E	24°C	7.0	-
	J100MAJ	28^0^40’42”N 76^0^27’18”E	26°C	6.5	-
	J101MAJ	28^0^39’49”N 76^0^26’54”E	24°C	6.6	-
	J102DUD	28^0^41’08”N 76^0^29’25”E	25°C	7.0	-
	J103DUD	28^0^40’55”N 76^0^29’04”E	25°C	7.2	-
	J104JHT	28^0^35’52”N 76^0^39’07”E	26°C	6.9	+
	J105SIL	28^0^32’52”N 76^0^41’07”E	24°C	7.1	-
	J106SIL	28^0^32’40”N 76^0^40’52”E	24°C	6.8	-
	J107SIL	28^0^32’53”N 76^0^40’45”E	25°C	7.2	-

^**#**^ **Sample was positive for *N*. *fowleri***

### Sequence analysis

**Fig 3:**
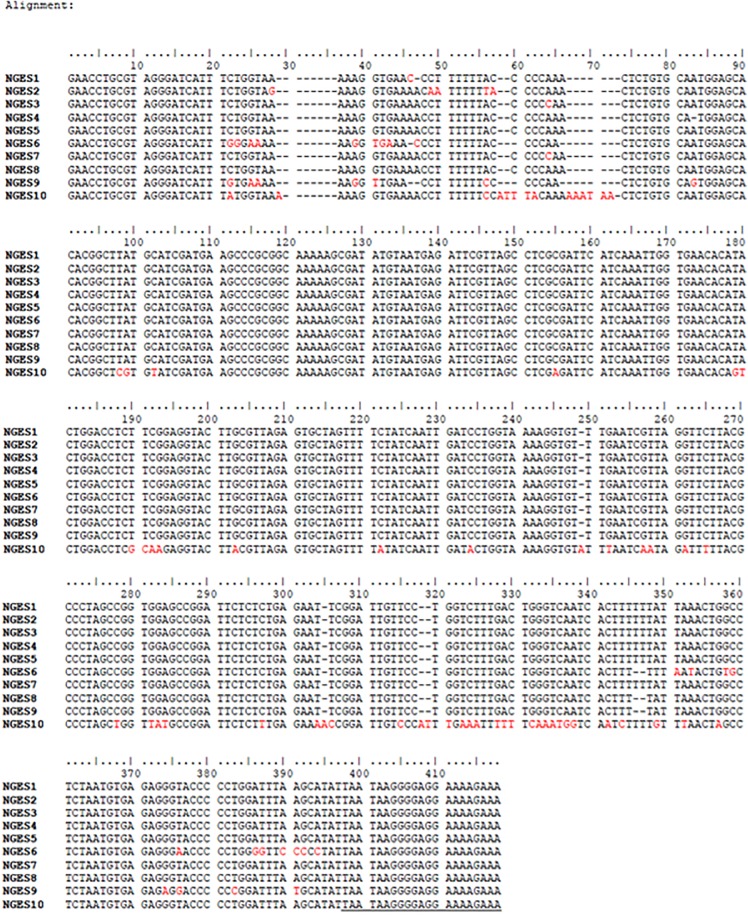
Multiple sequence alignment for amplicons NGES1-NGES10 using ClustalW program. Black colour dash (—-) indicates gap, red colour fonts base substitution or base insertion, forward and reverse primers are underlined.

The sequences of 10 randomly selected amplicons (ITS1-5.8S-ITS2) were analysed using ClustalW programme (Fig 3 and Table 2). Isolate no. 1 to 9 showed 99% homology with *Naegleria australiensis* (*N*. *australiensis*) except for isolate no. 3 and 9 which had shown 98% homology with *N*. *australiensis*. Isolate no. 10 had shown 99% homology with *N*. *fowleri*. The lengths (in bp) and differences in the ITS1, 5.8S, ITS2 and 28S rDNA sequences of representative isolates have been summarized in Table 3 and Fig 3.

**Table 2:** Result of DNA sequencing of the selected amplicons (NGES1-NGES10).

Isolate	Accession No.	Closest phylogenetic species	Maximum identity	Reference Strain Accession No.
NGES 1	KF700040	*Naegleria australiensis*	99%	GU597030
NGES 2	KF709528	*Naegleria australiensis*	99%	GU597034
NGES 3	KF709529	*Naegleria australiensis*	98%	GU597032
NGES 4	KF709530	*Naegleria australiensis*	99%	GU597035
NGES 5	KF709531	*Naegleria australiensis*	99%	GU597030
NGES 6	KF709532	*Naegleria australiensis*	*99%*	GU597031
NGES 7	KF709533	*Naegleria australiensis*	99%	GU597034
NGES 8	KF709534	*Naegleria australiensis*	99%	GU597039
NGES 9	KF709535	*Naegleria australiensis*	98%	GU597038
**NGES 10**	**KF709536**	***Naegleria fowleri***	**99%**	**AY033619**

**Table 3:** Length and position of base in the ITS-5.8S-ITS2-28S sequence of the selected isolates (NGES1- NGES10).

Isolate	Accession No.	Length and position of ITS1 sequence (in bp)	Length and position of 5.8S sequence (in bp)	Length and position of ITS2 sequence (in bp)	Length and position of 28S sequence (in bp)	Reference Strain Accession No.
NGES 1	KF700040	34(1–34)	174(35–209)	99(210–309)	44(310–354)	GU597030
NGES 2	KF709528	34(1–34)	174(35–209)	100(210–310)	44(311–355)	GU597034
NGES 3	KF709529	42(1–42)	173(43–216)	98(217–315)	44(316–360)	GU597032
NGES 4	KF709530	35(1–35)	172(36–208)	100(209–309)	44(310–354)	GU597035
NGES 5	KF709531	35(1–35)	173(36–209)	100(210–310)	44(311–355)	GU597030
NGES 6	KF709532	32(1–32)	173(33–206)	99(207–306)	43(307–350)	GU597031
NGES 7	KF709533	35(1–35)	173(36–209)	97(210–307)	47(308–355)	GU597034
NGES 8	KF709534	35(1–35)	173(36–209)	98(210–308)	44(309–353)	GU597039

### Phylogenetic analysis

**Fig 4:**
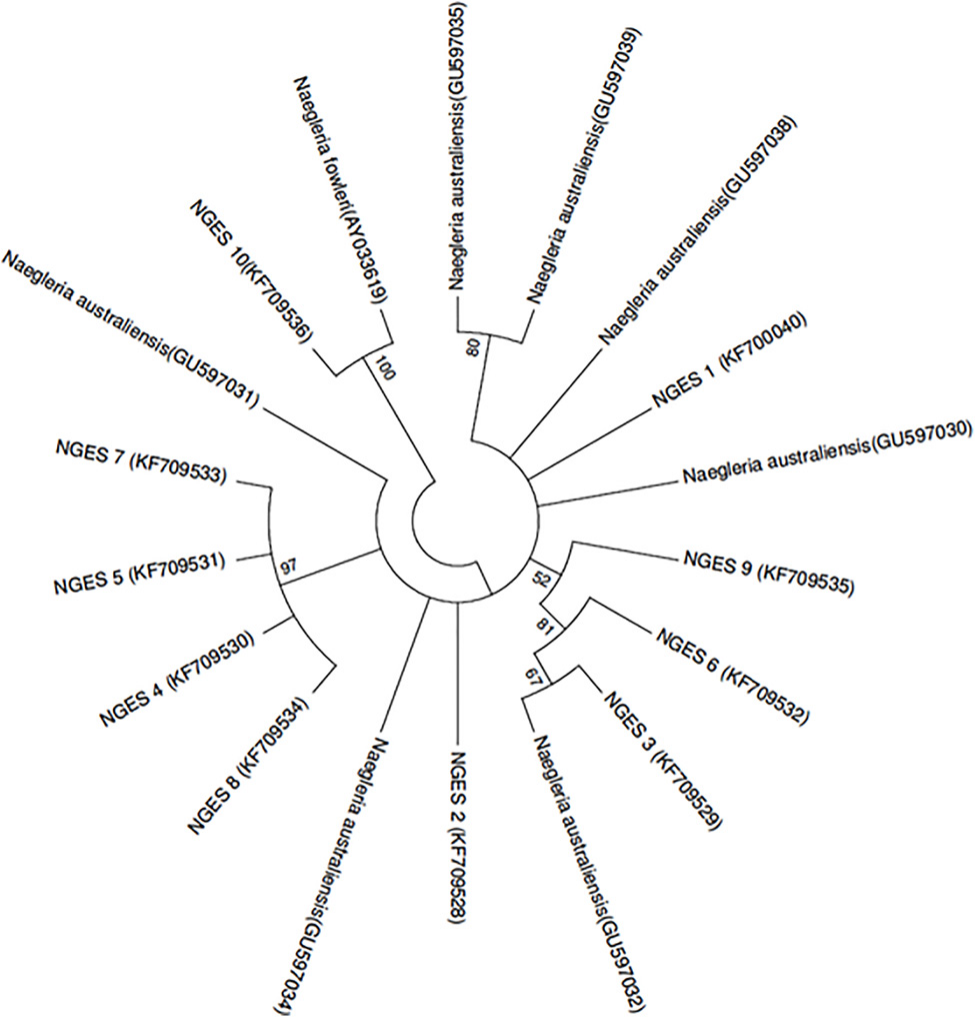
Neighbour-joining tree depicting the relationships between test isolates with amplicons (NGES1-NGES10) and reference strains of *Naegleria*. Numbers at the notes are percentage-bootstrapping values on 1000 replicates, and only values of > 50% are shown. GenBank accession numbers for reference sequences are indicated at the ends of the species designations.

The representative and reference isolates NGES 9, NGES 6, NGES 3 and *N*. *australiensis* GU597032 with similar sequences and length in the 5.8S (173 bp) were grouped together. NGES 4, NGES 5, NGES 7 and NGES 8 formed a clade to each other and showed similarity to the ITS1 (35bp). NGES 10 and *N*. *fowleri* (AY033619) formed a clade with each other showing similar sequences (Fig 4).

## Discussion and Conclusions

Present study has primarily focussed to detect and identify the presence of pathogenic *N*. *fowleri* in the different water bodies in Rohtak and Jhajjar of state Harayana. Initially, identification of all the isolates of *Naegleria -*like species was based on the examination of morphological forms of the organism. The trophozoites of all the isolates showed erupted pseudopodia or lobopodia that actively moved in unidirectional manner while the cysts had uniform smooth thick double walls. All theses isolate exhibited flagella in distilled water except for four isolates. The detection of the flagellate stage was one of the important indicators for differentiation of *Naegleria* species from that of other FLA. All the 43 isolates showed luxuriant growth at 37°C.

In this study we used the PCR based detection in order to confirm the *Naegleria* species that were present in our environment. *N*. *fowleri* culture obtained from American Type Culture Collection (ATCC no. 30894) was used as the positive control for the standardization of the PCR assay. Two primer sets i.e. the *N*. *fowleri* specific and *Naegleria* genus specific were designed from the ITS1-ITS2 regions as already described by Pélandakis et al. [44]**.** The ribosomal ITS sequence has been reported to be a useful tool for detecting both inter- and intra-species differences of various microorganisms, such as *Cryptosporidium parvum* [45], *Naegleria* spp. [46] and *Vahlkampfia* spp. [47]. Within *Naegleria* spp. the differences between genus (inter-species) and species (intra-species of *N*. *fowleri*) are due to the sequence polymorphisms that occur at the ITS2 and ITS1 regions [40]. The ITS region has also been used for the identification of the new *Naegleria* isolates [11, 44].

In the current study *Naegleria* genus specific primer set successfully amplified the DNA template from 37 isolates, while the species specific primer set could amplify only one isolate of *N*. *fowleri* out of 37 isolates. The species specific primer set produced the amplicon consisting of 308bp, while the genus specific primer set produced amplicon consisting of 408–410 bp. Upon sequence analysis, *N*. *fowleri* (NGES10) identified in the study belonged to Type 2. Presence of the Pathogenic *N*. *fowleri* has been detected on all continents, except in Antarctica. So far, seven types have been reported in Europe, three in American and two in Asian continents [48]. Both type 3 and type 2 were common to Asian, American and European continents [19].

**Fig 5:**
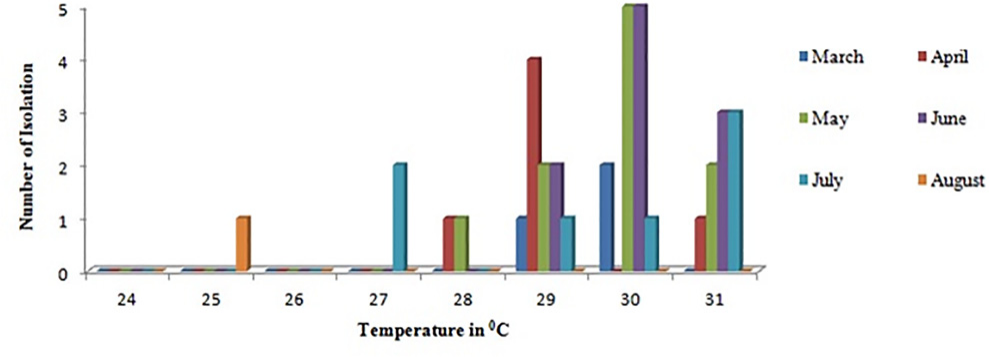
Graph depicting the association between temperature of the sampling sites and isolation frequency of *Naegleria* spp. Y axis denotes the number of isolation and X axis denotes minimum and maximum temperature range of the sampling sites during the entire season (March to August).

The other most frequently isolated species in this study was *N*. *australiensis*. It is pathogenic in mice but has not been isolated from human cases [49]. *N*. *australiensis* is also the closest phylogenetic relative of *N*. *fowleri* (Fig 4) other *than N*. *lovaniensis* [1, 19]. It has been reported that *N*. *lovaniensis* is usually the most dominant species of *Naegleria* in warm water [50]. The data in the present study reveals that *N*. *australiensis* is the most frequently encountered species in the northern part of India especially Haryana. The reason for this feature observed warrants further investigation. *Naegleria* spp. were most frequently isolated during the summer season (April to July) (Fig 5), with the onset of monsoon the isolation rate reduced. The isolation frequency was high for water bodies with temperature ranging between 29–31°C (Table 1 and Fig 5). These were the noteworthy findings in the study.

In summary, our study is the first to use a PCR-based approach to screen and document the presence of *Naegleria* spp., in a variety of water bodies in the Northern state of India. Our results provide the evidence that *N*. *fowleri* is present in these natural water bodies. These FLA poses health risks to those people who use these aquatic systems for recreational activity and their day to day use. Considering the limited understanding of the ecology of *N*. *fowleri* in India, practical measures have to be taken for prevention and control of *Naegleria* infections. It includes better awareness of the disease within the medical community and educating general public with the help of civic authorities.
